# Mid- to long-term results of total disc replacement for lumbar degenerative disc disease: a systematic review

**DOI:** 10.1186/s13018-018-1032-6

**Published:** 2018-12-26

**Authors:** Xu-Dong Cui, Hai-Tao Li, Wen Zhang, Lin-Lin Zhang, Zong-Ping Luo, Hui-Lin Yang

**Affiliations:** 1 Orthopedic Institute, Soochow University, Suzhou, 215006 Jiangsu China; 2 the First Affiliated Hospital, Soochow University, Suzhou, 215006 Jiangsu China

**Keywords:** Lumbar total disc replacement, Mid- to long-term follow-up, Systematic review

## Abstract

**Background:**

Lumbar total disc replacement (TDR) has shown satisfactory clinical outcomes with few complications and reoperations at short-term follow-up, but the mid- to long-term results are not clear.

**Purpose:**

The objective of this study was to evaluate the mid- to long-term clinical outcomes of artificial TDR for lumbar degenerative disc diseases.

**Patients and methods:**

A systematic search was conducted using the PubMed database to identify studies of TDR surgery that included at least 3 years of follow-up. The search keywords were as follows: lumbar, total disc replacement, and arthroplasty. The following data were extracted: patient demographics, visual analogue scale (VAS) and Oswestry disability index (ODI) scores, satisfactory rate, clinical success rate, complications, and reoperations.

**Results:**

Thirteen studies, including eight prospective studies and five retrospective studies, met the criteria. A total of 946 patients were identified who reported at least 3 years of follow-up results. The artificial prostheses in these studies were ProDisc-L, Charité, AcroFlex, Maverick, and XL TDR. Patients with lumbar TDR demonstrated significant improvements in VAS scores of 51.1 to 70.5% and of − 15.6 to − 44.4 for Oswestry disability index (ODI) scores at the last follow-up. Patient satisfaction rates were reported in eight studies and ranged from 75.5 to 93.3%. Complication rates were reported in 11 studies, ranging from 0 to 34.4%. The overall reoperation rate was 12.1% (119/986), ranging from 0 to 39.3%, with eight of the 13 studies reporting a reoperation rate of less than 10%.

**Conclusions:**

This review shows that lumbar TDR effectively results in pain relief and an improvement in quality of life at mid- to long-term follow-up. Complication and reoperation rates were acceptable. However, this study did not provide sufficient evidence to show that lumbar TDR is superior to fusion surgery. To answer that question, a greater number of high-quality randomized controlled trials (RCTs) are needed.

## Background

The objective of this study was to evaluate the mid- to long-term clinical outcomes of artificial total disc replacement (TDR) for lumbar degenerative disc diseases. Degenerative disc disease is one of the main triggers of severe low back pain and sciatica, which are indications for surgery. Re-establishing spinal stability is the key for achieving patient recovery and long-term therapeutic outcomes. Therapy can be divided into two groups: fusion [6] and non-fusion [4, 35] surgery. The most commonly used surgical technique for re-establishing spinal stability is fusion, which has been identified as the gold standard for the treatment of lumbar degenerative disc disease. However, it has intrinsic drawbacks: it sacrifices the motion of vertebral segments and changes the biomechanics of the spine, potentially causing adjacent segment disease or pseudoarthrosis [12, 14, 16, 19]. To restore spinal motion and overcome the shortcomings of spinal fusion surgery, lumbar TDR was conceived to restore the function of the intervertebral discs.

Following the satisfactory clinical outcomes of the early artificial discs, the SB Charité and ProDisc-L [3, 10] prostheses and many additional LTDR prostheses have been designed and used clinically. From the nationwide inpatient sample (NIS) data between 2000 and 2009, even though clinical use of the Charité and ProDisc-L prostheses had been approved by the FDA, TDRs accounted for only 2.7% of the surgical treatments for lumbar degenerated disc disease during those years [48]. Although several published articles with 2 years of follow-up reported that lumbar TDR had superior clinical outcomes with fewer complications and a lower rate of reoperation than fusion surgery [30, 46], the clinical use of lumbar TDR is still at a low level [21]. Common reasons for the low usage include the lack of long-term efficacy studies, the unfamiliarity of spinal surgeons with the technique, the existence of clinical complications and revision surgeries, conflicting data from published studies, and the absence of health insurance support [2, 37, 45].

The purpose of this study was to systematically review the clinical efficacy and safety of lumbar TDR at mid- to long-term follow-up. In this study, mid- to long-term follow-up studies of lumbar TDR were reviewed through the PubMed database, and the clinical effectiveness, complications, and rate of reoperations were selected for analysis to provide more information to support greater potential clinical utilization.

## Methods

### Search strategy and inclusion criteria

**Fig. 1:**
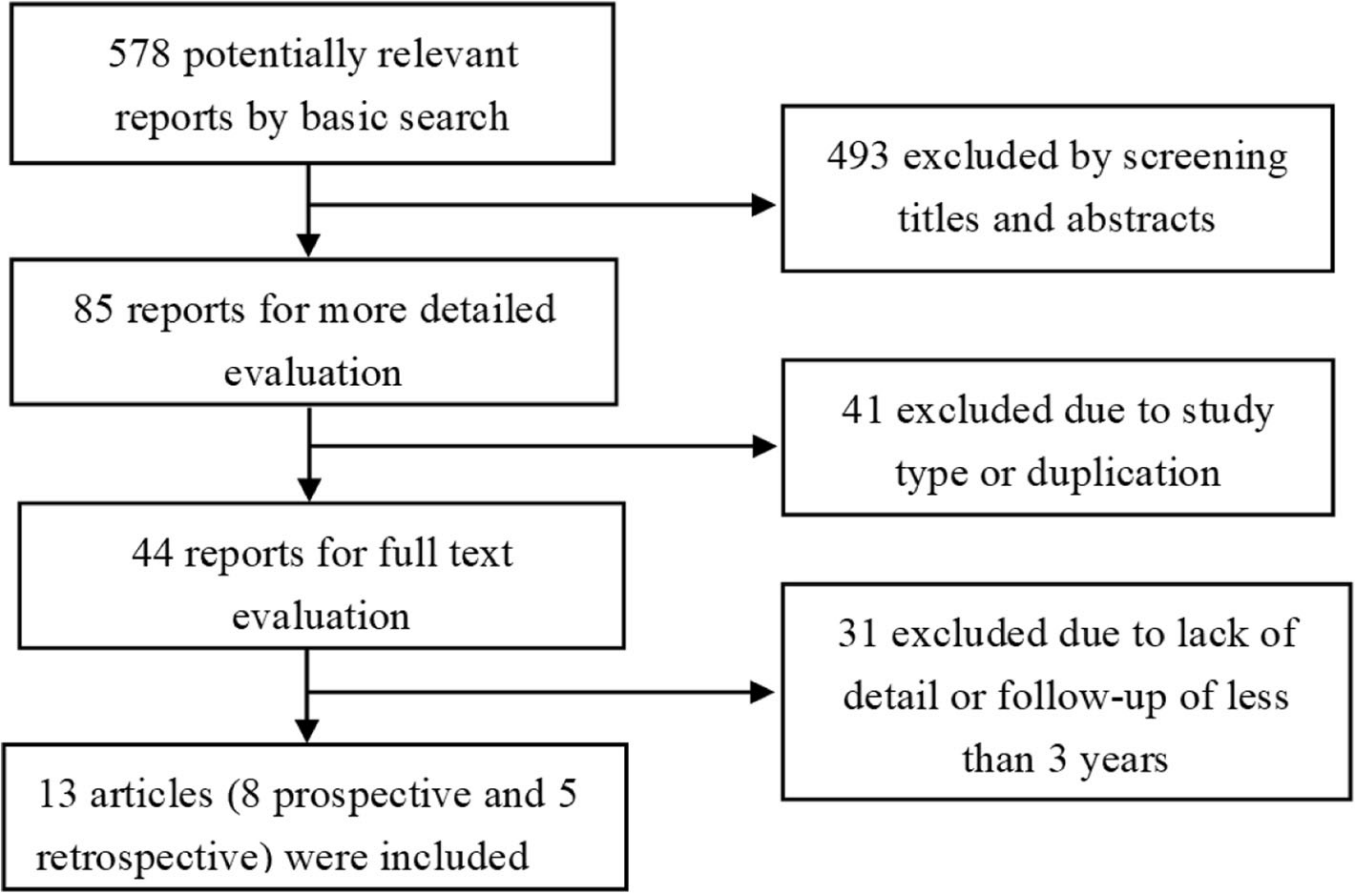
The flow chart of study selection

The search was conducted using the PubMed database to identify cases of TDR surgery with at least 3 years of follow-up. Only articles in English-language journals or published with English abstracts were included, with no limits on publication date. Search keywords were as follows: lumbar, total disc replacement, and arthroplasty. To conduct a comprehensive analysis of lumbar disc replacements, we performed a broad search of articles, including both retrospective and prospective cohort studies. The search results and strategy are shown in Fig. 1. Two researchers were involved in the selection and screening of the relevant literature. Studies included met the following criteria: prospective and retrospective studies of lumbar TDR in the treatment of lumbar degenerative disease; at least 3 years postoperative follow-up; and the clinical prognosis index meeting at least one of the following: visual analogue scale (VAS) of back and/or leg pain, the Oswestry disability index (ODI), and complications or reoperations. The exclusion criteria were as follows: studies in which hybrid constructs were used; spinal infection, trauma, or tumor present before lumbar TDR surgery; and non-prospective or retrospective studies, such as case reports and reviews.

### Data extraction

From the included articles, the following data were extracted: patient demographics, study design, follow-up duration and follow-up rate, type of artificial disc, clinical prognosis, complications, and reoperations. Clinical prognosis included the VAS, ODI, clinical success rate, and satisfaction rate. To maintain the unity of the data, the VAS and ODI were processed further as the VAS improvement rate and ODI improvement score. The VAS improvement rate was defined as (postoperative mean VAS score − preoperative mean VAS score)/(0 − preoperative mean VAS score) × 100%. The ODI improvement score was defined as the postoperative mean ODI − preoperative mean ODI. Complications were subdivided into three subgroups: surgical approach-related or intraoperative complications, device-related complications, and postoperative complications. Surgical approach-related or intraoperative complications included hematoma, hernia, vessel injury, dura mater injury, urinary injury, retrograde ejaculation, or sympathetic injury. Device-related complications included device subsidence, implant displacement, and device failures. Postoperative complications included segmental or adjacent degeneration, neurological symptoms, deep venous thrombosis, or persistent symptoms of low back pain.

### Quality assessment

Two authors independently assessed the quality of the studies included in the review using the Oxford Levels of Evidence criteria [47]. The level of evidence (I–V) was assessed for each article according to published criteria. The Assessment of Multiple Systematic Reviews (AMSTAR) instrument was used as an additional methodological quality tool, providing a measure of reliability, validity, and responsibility. The AMSTAR tool consisted of 11 components [38], and each component had four different answers, i.e., 1 point for “yes,” 0 point for “no,” “cannot answer,” or “not applicable.”

## Results

### Description of included studies and quality assessment

From the systematic search, a total of 578 references were identified. Of these, 493 were excluded because they were not relevant to this review after screening the title and abstract, and 41 were excluded because they involved duplication or did not meet the study type criteria. On evaluating the full text of the remaining 44 references, 31 were excluded because they lacked the required details or had less than 3 years of follow-up. Finally, 13 references were evaluated in detail using the search strategy summarized in Fig. 1. Of the 13 articles, eight were prospective studies, five were retrospective cohort studies, and three of the articles compared TDR with fusion with at least 3 years of follow-up. According to the Oxford Levels of Evidence quality assessment criteria of the included studies, three studies were evaluated as level I, five studies were at level II, and five studies were at level III. The AMSTAR score was 6 points for the 11 questions (Table 1), which is considered medium quality.

**Table 1:** AMSTAR evaluation form

Questions	Answers
1. Was an “a priori” design provided? The research question and inclusion criteria should be established before the conduct of the review.	Yes
2. Was there duplicate study selection and data extraction? There should be at least two independent data extractors and a consensus procedure for disagreements should be in place.	Yes
3. Was a comprehensive literature search performed? At least two electronic sources should be searched. The report must include years and databases used (e.g., Central, EMBASE, and MEDLINE). Key words and/or MESH terms must be stated, and where feasible, the search strategy should be provided. All searches should be supplemented by consulting current contents, reviews, textbooks, specialized registers, or experts in the particular field of study, and by reviewing the references in the studies found.	No
4. Was the status of publication (i.e., grey literature) used as an inclusion criterion? The authors should state that they searched for reports regardless of their publication type. The authors should state whether or not they excluded any reports (from the systematic review), based on their publication status, language etc.	No
5. Was a list of studies (included and excluded) provided? A list of included and excluded studies should be provided.	Yes
6. Were the characteristics of the included studies provided? In an aggregated form such as a table, data from the original studies should be provided on the participants, interventions, and outcomes. The ranges of characteristics in all the studies analyzed e.g., age, race, sex, relevant socioeconomic data, disease status, duration, severity, or other diseases should be reported.	Yes
7. Was the scientific quality of the included studies assessed and documented? “A priori” methods of assessment should be provided (e.g., for effectiveness studies if the author(s) chose to include only randomized, double-blind, placebo controlled studies, or allocation concealment as inclusion criteria); for other types of studies, alternative items will be relevant.	Yes
8. Was the scientific quality of the included studies used appropriately in formulating conclusions? The results of the methodological rigor and scientific quality should be considered in the analysis and the conclusions of the review, and explicitly stated in formulating recommendations.	No
9. Were the methods used to combine the findings of studies appropriate? For the pooled results, a test should be done to ensure the studies were combinable, to assess their homogeneity (i.e., chi-squared test for homogeneity, *I*^2^). If heterogeneity exists, a random effects model should be used and/or the clinical appropriateness of combining should be taken into consideration (i.e., is it sensible to combine?).	Not applicable
10. Was the likelihood of publication bias assessed? An assessment of publication bias should include a combination of graphical aids (e.g., funnel plot, other available tests) and/or statistical tests (e.g., Egger regression test, Hedges-Olken).	Not applicable
11. Was the conflict of interest included? Potential sources of support should be clearly acknowledged in both the systematic review and the included studies.	Yes

### Study characteristics

**Fig. 2:**
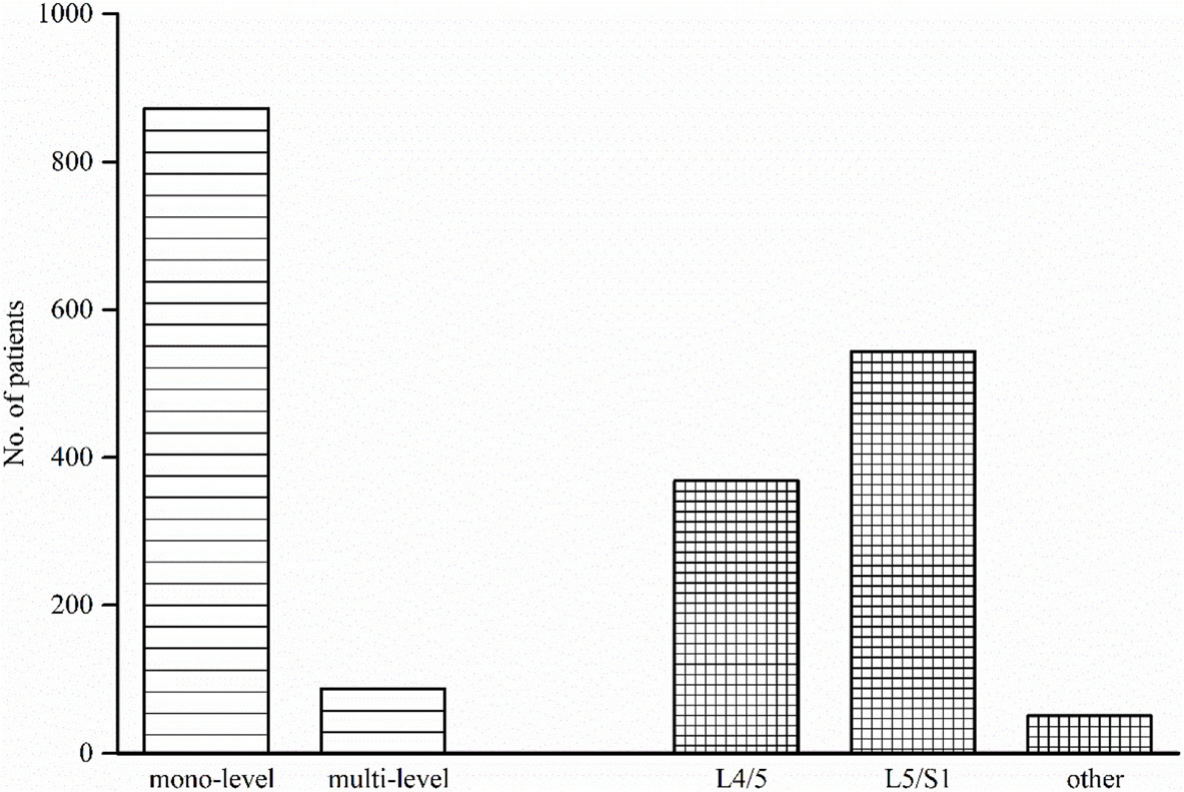
The distribution of the segment surgery level. In the studies of Sköld et al. [41] and Lu et al. [23], surgery level was not subdivided and was not included in this implant distribution figure

The characteristics of the reviewed studies analyzed are presented in Table 2. In all the studies included in the review, 946 patients were followed up, of whom 666 and 280 patients were from prospective and retrospective studies, respectively. The follow-up rate ranged from 74.6 to 100%; the minimum and maximum ages of the studied populations were 19 and 79 years old, respectively. The mean follow-up time ranged from 3.0 to 17.3 years, with at least 3 years of follow-up. The artificial prostheses included were ProDisc-L, Charité, AcroFlex, Maverick, and XL TDR. A total of 1048 prostheses were implanted, single-segment TDRs were performed on 872 patients, and multi-segment TDRs were performed on 88 patients (Fig. 2). A total of 369 prostheses were implanted into level L4/L5, 543 prostheses were implanted into level L5/S1, and 51 were implanted into other segments.

**Table 2:** The characteristics of the patients including the selected analysis

Study	Year	Type of study	Type of prosthesis	Number of patients (T/F)	FU rate (%)	Mean age (min, max)	Mean FU years	Evidence level
Guyer et al. [17]	2012	Prospective	Charité	90/90	100	40.0 (19–60)	5 (N/A)	I
Zigler and Delamarter[50]	2012	Prospective	ProDisc	126/161	78	38.7 (N/A)	85.1% (N/A)	I
Van De Kelft and Verguts [43]	2012	Prospective	Maverick	45/50	90	37.1 (N/A)	4 (N/A)	II
Meir et al. [27]	2013	Prospective	AcroFlex	23/28	82.10	41 (30–54)	9.6 (8.7–11.3)	II
Sköld et al. [41]	2013	Prospective	Charité, ProDisc, Maverick	80/80	100	40.2 (21/55)	5 (N/A)	I
Siepe et al. [39]	2014	Prospective	ProDisc	181/201	90	43.0 (21.9–66.1)	7.4 (5.0–10.8)	II
Tohmeh and Smith. [42]	2015	Prospective	XL TDR	64/64	100	45.3 (26–67)	3.0 (N/A)	II
Laugesen et al. [20]	2017	Prospective	ProDisc	57/68	84	49.6 (34.5–79.0)	10.6 (8.1–12.6)	II
Putzier et al. [34]	2006	Retrospective	Charité	53/71	74.60	44 (30–59)	17.3 (14.5–19.2)	III
David [7]	2007	Retrospective	Charité	106/108	98	36.4 (23–50)	13.2 (10.0–16.8)	III
Park et al. [29]	2012	Retrospective	ProDisc	35/35	100	46.5 (27–70)	6.0 (5.0–7.8)	III
Lu et al. [23]	2015	Retrospective	Charité	32/35	91.40	41.1 (28.6–51.3)	11.8 (11.3/13.8)	III
Park et al. [31]	2016	Retrospective	ProDisc	54/64	84.40	44.1 (29–59)	10.0 (5.1–12)	III

*NO* number, *T* total, *F* follow, *FU* follow-up, *min* minimum, *max* maximum, *N/A* not available

### Clinical outcomes

In the studies included in the review, clinical effectiveness was mainly evaluated by pain scores, ODI [28], activity or work status, segmental ROM, clinical success rate, and patient satisfaction after lumbar TDR, as shown in Table 3. Pain measurements were mainly assessed using the VAS, back and leg pain scores. Data calculated from the nine studies that used pain scores showed that the rate of improvement ranged from 51.1 to 83.5%. ODI was another important measure of lumbar function and was used in eight studies. Improvements of 15 or more points are commonly used as one of the criteria for clinical success [17, 29, 31, 34, 39, 41–43]. The mean improvement in ODI score from preoperative to the last follow-up was in the range of − 15.6 to − 44.4 points. The clinical success rate was reported in six studies, ranging from 53.3 to 87.2% and basically used the same evaluation criteria. Satisfactory clinical outcomes were reported in eight studies, with outcomes recorded as excellent or good at the last follow-up ranging from 75.5 to 93.3%. Work status and sports activity, used as measures of functional recovery, were recorded in seven studies. All these studies recorded that at least 65.9% of patients had part- or full-time employment following TDR surgery.

**Table 3:** Clinical outcomes at last follow-up of the included studies

Study	Type of prosthesis	VAS improvement rate (%)	ODI score improvement	Clinical success rate (%)	Satisfaction rate (%)
Guyer et al. [17]	Charité	55.7	− 21.5	57.8	78.0
Zigler and Delamarter [50]	ProDisc	51.1	− 44.4	53.7	NA
Van De Kelft and Verguts. [43]	Maverick	61.0	− 28.55	63.0	77.0
Meir et al. [27]	AcroFlex	NA	− 15.6	NA	NA
Sköld et al. [41]	Charité, ProDisc, Maverick	63.6	− 24.6	72.5	79.0
Siepe et al. [39]	ProDisc	54.9	NA	NA	86.3
Tohmeh and Smith [42]	XL TDR	NA	NA	NA	93.3
Laugesen et al. [20]	ProDisc	52.9	NA	NA	NA
Putzier et al. [34]	Charité	NA	NA	NA	75.5
David [7]	Charité	NA	NA	NA	NA
Park et al. [29]	ProDisc	70.5	− 25.1	71.4	88.6
Lu et al. [23]	Charité	82.8	− 28.2	87.5	NA
Park et al. [31]	ProDisc	57.0	− 19.1	66.7	87.2

VAS improvement rate = (postoperative mean VAS score − preoperative mean VAS score)/(0 − preoperative mean VAS score) × 100%

ODI improvement score = postoperative mean ODI − preoperative mean ODI

### Complications and reoperations

Among the 13 included studies, 10 studies reported complication rates ranging from 0 to 34.4%. The other three studies by Meir et al. [27], Putzier et al. [34], and Tohmeh and Smith [42] did not mention the complication rate in their reports, which were analyzed separately. Meir et al. [27] reported that of 23 patients treated with the prosthesis of AcroFlex, four patients suffered disabling pain after surgery (two osseointegrate, one facet joint arthropathy, and one no obvious cause), and seven patients had revision surgery due to implant failure. At their 10-year follow-up, 12/14 AcroFlex levels from the 11 non-revision patients showed heterotopic bone formation, including 7/14 levels regarded as “severe.” For AcroFlex prostheses, due to device failure and the reoperation rate being much higher than with other prostheses, further implantation of the AcroFlex prosthesis was terminated. Putzier et al. [34] reported the longest follow-up, exceeding 17 years; a total of 53 patients were included, no intraoperative complications were reported, 12 patients (23%) had a segmental fusion due to implant failure or pain, and 32 patients showed ossifications resulting in spontaneous ankylosis. In the study by Tohmeh and Smith [42], 64 patients were followed up for 3 years, and no intraoperative complications were reported. Immediate complaints after the operation were mainly hip flexion weakness in 15.6% (10/64), lower extremity motor deficits in 10.9% (7/64), and sensory deficits along the lower extremities in 15.6% (10/64). All of these complications were improved before the last follow-up, and no reoperation was performed. In the remaining 10 articles, 841 patients were included with a total complication rate of 15.9% (134/841). Through summary and analysis, the complications were divided into three separate groups: surgical approach and intraoperative-related, device-related, and postoperative complications (details in Table 4).

**Table 4:** Complications in the included studies

Study	Type of prosthesis	Complication (rate)	Surgical approach- or intraoperative-related	Device-related	Postoperative
Guyer et al. [17]	Charité	20/90 (22.2%)	0	1 Device subsidence 2 Early postoperative implant displacement	13 Neurological deterioration 1 Symptomatic spondylolisthesis at L5 2 Facet degeneration 1 Unknown
Zigler and Delamarter [50]	ProDisc	16/161 (9.9%)	1 Technique error with inlay was inserted backward 2 Retrograde ejaculation 1 Clinical significant blood loss	2 Polyethylene migration due to extreme trauma 1 Polyethylene inlay migration 1 Implant migration	6 Unresolved pain 1 Nerve root compression 2 DVT
Van De Kelft and Verguts [43]	Maverick	2/45 (4.4%)	2 Vein injury	0	0
Sköld et al. [41]	Charité, ProDisc, Maverick	13/80 (16.3%)	2 Hematoma 2 Wound hernia	1 Subsidence	6 Suspected facet joint pain 1 Nerve entrapment 1 Meralgia paresthestica
Siepe et al. [39]	ProDisc	26/181 (14.4%)	1 Abdominal hematoma 1 Retroperitoneal hematoma 1 Retroperitoneal lymphocele 4 Retrograde ejaculation 4 Postsympathectomy syndrome 1 Plexus hypogastricus superior lesion with sexual dysfunction 1 Persisting retroperitoneal secretion 1 Primary suboptimal implant placement 1 Intraoperative posterior wall fracture and posterior fragment dislocation	2 Subsidence 2 Inlay dislocation and subluxation of prosthesis 1 Implant dislocation following fall 2 weeks postoperatively 1 Split fracture L4 following TDR at L4–5	3 Adjacent level disc herniation 1 Index segment spinal stenosis 1 Postoperative neuropathy L5 1 CVA with bilateral isthmus stress fracture and subluxation of TDR 1 Bilateral isthmus stress fracture 1 DVT+PE+lysis
Laugesen et al. [20]	ProDisc	19/57 (33%)	0	0	14 Back pain and or radiculopathy 1 Spondylolysis and spondylolisthesis 3 Facet arthrosis 1 Unknown
David [7]	Charité	22/106 (20.8%)	0	2 Early core subluxation 1 Late core failure 3 Subsidence	5 Symptomatic facet arthrosis 1 Continued axial low back pain 1 Sciatica with drop foot 4 Partial device ossification 2 Complete ossification 2 Disc herniation 1 Spinal stenosis
Park et al. [29]	ProDisc	0/35 (0%)	0	0	0
Lu et al. [23]	Charité	11/32 (34.4%)	2 Illac vein injury 2 Anhidrosis 2 Abdominal hernia	3 Subsidence	1 Pedicle fracture 1 Severe leg pain
Park et al. [31]	ProDisc	5/54 (9.3%)	0	0	2 Adjacent spondylolisthesis 1 Index level instability 2 Index level facet arthritis

In the study of Meir et al. [27], Putzier et al. [34] and Tohmeh and Smith [42]*,* complication rate were not mentioned, this three studies were not included in this table

In this review, the total rate of reoperation was 119/986 (12.1%), with the largest rate of 39.3% reported by Meir et al. [27], who used the AcroFlex prosthesis. The reoperation rate ranged from 0 to 39.3%, with eight out of 13 studies reporting rates of less than 10%. Only two studies reported reoperation rates higher than 30: Meir et al. using the AcroFlex prosthesis (39.3%) and Laugesen et al. (33%). The reoperation time was recorded in six studies, with a mean of 0.8–6.9 years after TDR surgery. The indications for reoperation and surgical methods are summarized in Table 5.

**Table 5:** Reoperations of the included studies

Study	Reoperation rate	Reoperation time			Indication for reoperation	Reoperation surgery
		Mean time years	≤ 2 years	> 2 years		
Guyer et al. [17]	7/90 (7.8%)	–	5	2	1 Symptomatic spondylolisthesis 1 Device subsidence with back pain 2 Facet degeneration 2 Early postoperative implant displacement	6 Supplemental fixations 1 Without internal fixation
Zigler and Delamarter [50]	11/161 (6.8%)	–	6	5		
Van De Kelft and Verguts [43]	0/45 (0%)	–	–	–	–	–
Meir et al. [27]	11/28 (39.3%)	3.8 (1.9–8.3)	–	–	7 Implant failure 4 Ongoing disabling pain	2 Supplemental fixation without implant removed 9 Implant removed
Sköld et al. [41]	16/80 (20%)	–	–	–	9 General reoperation 9 Device-related reoperation 2 New operation at new level TDR 3 Posterior lateral fusion 1 Decompression 1 Disc hernia	
Siepe et al. [39]	29/181 (16.0%)	0.8 (0–4.6)	–	–	9 Device- or technique related-complications 4 General surgery-related reoperation 10 Persisting symptoms of low back pain 4 Adjacent segment pathologies	4 General revision surgery 4 Posterior instrumentation 2 Microsurgery decompression 2 Anterior revision with implant replacement 1 Anterior revision with implant removal 2 Posterior fusion and cement augmentation 3 Postoperative discectomy and adjacent level fusion
Tohmeh and Smith [42]	0/64 (0%)	–	–	–	–	–
Laugesen et al. [20]	19/57 (33%)	3.5 (0.5–8.8)			14 Back pain and or radiculopathy 1 Spondylolysis and spondylolisthesis 3 Facet arthrosis 1 Unknown	N/A
Putzier et al. [34]	5/53 (9.4%)	–	–	–	7 Implant fracture 3 Implant subsidence 1 Implant dislocation 1 Persisting pain due to progressive degeneration	
David [7]	14/106 (13.2%)	5.0 (0–12)	4	9		8 Index fusion procedure 3 Prosthesis replacement 2 Adjacent microdiscectomy 1 Decompression and fusion
Park et al. [29]	0/35 (0%)	–	–	–	–	–
Lu et al. [23]	2/32 (6.3%)	4.3 (1.7–7.0)	1	1	1 Unclipping of the UHMWPE insert 2 Retroperitoneal hematoma	–
Park et al. [31]	5/54 (9.3%)	6.9 (4.5–9.3)	0	5	1 Degenerative spondylolisthesis at adjacent level 1 Degenerative spondylolisthesis at adjacent level and facet arthritis at index level 1 Suspicious instability at index level 2 Facet arthritis at index level	5 PLIF

## Discussion

Total disc replacement aimed to preserve the motion of the affected segments and to prevent adjacent degeneration. In recent decades, several artificial discs have been designed and used in cervical or lumbar segment and got satisfactory clinical effect in early reports; however, both were lack of prospective randomized trials with long-term follow-up to evaluate the effectiveness, safety, and complication after artificial discs implanted [11]. Biomechanically, lumbar TDR and cervical TDR have the same principle and device structure in the management of spinal motion, and both used an anterior approach, but with more complex anatomy in the lumbar region, there are more limited indications and higher complication rates in lumbar TDR [11, 36].

Lumbar artificial discs were first designed as a type of steel ball by Fernström in 1960 [13], which proved to be a failure as it subsided into the subchondral bone. In the 1980s, Schellnack and Büttner-Janz first reported SB Charité prosthesis, and later, Marnay reported ProDisc-L prosthesis. These two types of prostheses initiated the onset of the clinical use of lumbar artificial discs. Since then, different materials and designs of artificial discs have been developed for clinical therapy. A previous meta-analysis on the 2-year follow-up outcomes showed that, compared with fusion surgery, lumbar TDR has a slightly better clinical outcome and fewer complications and reoperations, but the mid- to long-term results of lumbar TDR are not clear [30]. This is the first systematic review of lumbar TDR clinical efficacy and safety of mid- to long-term follow-up outcomes. In this systematic review, eight prospective studies and five retrospective studies were included, involving the most common prostheses, i.e., Charité, ProDisc, Maverick, AcroFlex, and XL TDR.

### Clinical efficacy of lumbar total disc replacement

VAS and ODI scores are the most frequently used scales to evaluate the clinical efficacy of lumbar TDR prostheses. Ostelo et al. [28] studied the minimum significant change in clinical improvement in measures of VAS and ODI scores for low back pain and showed that a 30% change from baseline would be considered clinically meaningful. In the included studies, eight showed significant improvements in VAS and ODI scores at the last follow-up, which indicated that TDR could effectively alleviate pain and enhance quality of life. As mentioned by Siepe et al. [39], although overall VAS and ODI scores were significantly improved from baseline at the final follow-up of 7.4 years, VAS scores slightly deteriorated from 2.6 to 3.3 at 48 months after TDR surgery. Although the clinical outcomes of VAS and ODI scores might very slowly deteriorate with the increase in postoperative time, a much better clinical outcome than for the preoperative conditions was shown in this study.

The satisfaction rate is one of the essential measurements of clinical outcomes because it represents a patient’s subjective feelings. Eight studies reported a high satisfaction rate in the range of 75.5 to 93.3%, and the high satisfaction rate gave the patients the confidence to choose the same surgery again. In the study of Park et al. [29], 88.6% of patients were either “satisfied” or “somewhat satisfied,” of which 60% were willing to undergo the same treatment again. Despite the high satisfaction rate, patients dissatisfied during follow-up should not be ignored. As shown in the Sköld et al. [41] study, although the clinical outcome had only a small degree of deterioration, 11.25% of patients remained unsatisfied and reported that they had deteriorated in comparison with their preoperative status. However, the reasons for this phenomenon were not mentioned. Another study by Siepe et al. [39] demonstrated a similar situation, where VAS scores slightly deteriorated from 2.6 to 3.3 during follow-up; 13.7% of patients reported dissatisfaction with their outcomes, despite their VAS scores being well below the preoperative baseline. This dissatisfaction with the treatment might be caused by many factors that need further analysis.

The clinical success rate was reported in six studies and ranged from 53.7 to 71.4%, as shown in Table 3. This success rate is a synthetic index used to evaluate the efficiency of lumbar TDR. This criterion, which is not validated by the FDA, is defined as a combination of successful outcomes: ODI improvement of at least 15 points from baseline; significant pain relief, maintenance, or improvement in neurologic status; and no severe device adverse events or device-related reoperation. In the Guyer et al. [17] study, the clinical success rate was 65.2% at 2-year follow-up and 57.8% at 5-year follow-up, and this success rate was not less than that of the BAK group in their study. In the Van de Kelft and Verguts [43] study, the postoperative clinical success rate achieved at 24 months was 75.5% vs 63% at 48 months. The decline in the success rate was related to ODI improvement of less than 15 points and decreased pain reduction. Park et al. [31] used a higher standard of at least 25% improvement in ODI score as the criterion for clinical success and reported a 76.9% clinical success rate for the good candidates group and only 40.0% for the bad candidates group. Zigler and Delamarter [50] reported a statistical success rate of 53.7% using modified criteria that included the parameters of ROM success. Although their statistical success rate was the lowest in the six studies, compared with the fusion group, the TDR groups demonstrated statistical noninferiority. Overall, the clinical success rate as a comprehensive index showed that lumbar TDR had a high clinical success rate and was not inferior to fusion surgery.

### Complications and reoperations of lumbar total disc replacement

The complication and reoperation rates of lumbar TDR surgery have always been of great concern. Previous studies have reported complication rates and reoperation rates in the range of 1 to 17.5% with rates of 2.3 to 10% at short-term follow-up [1, 8, 9, 15, 49], respectively. In a meta-analysis of RCTs assessing TDR versus fusion in the treatment of lumbar degenerative disc disease at 2 years follow-up, Wei et al. [46] indicated that the TDR group had a lower complication rate than the fusion group, and the reoperation rate was not clinically significantly different between the two groups. Although previous studies reported satisfactory clinical outcomes, the complications and reoperations over a long period of implantation require clarification to gauge the safety of lumbar TDR. Among the included studies, Meir et al. [27] reported the largest reoperation rate of 39.3%, including seven device failures and four incidents of disabling pain at 10 years follow-up. Due to the much higher device failure and reoperation rates compared to those associated with other prostheses, further implantation of the AcroFlex prosthesis was terminated. As shown in Table 4, the incidence of complications ranged from 0 to 33%, and the reoperation rate ranged from 0 to 39.3%.

In recent decades, lumbar TDR prostheses have been implanted in a considerable number of patients, despite enormous improvements in prosthesis size, surgical technique, and patient selection, complications still occur. A comprehensive description and analysis of the complications is important. In this review, complications were classified into three groups: surgical approach-related or intraoperative-related complications, device-related complications, and postoperative complications. Surgical approach-related or intraoperative-related complications were mainly hematoma, incisional hernia, retrograde ejaculation, vein injury, or urinary injuries. Device-related complications mostly resulted from polyethylene inlay migration or implant dislocation. Postoperative complications, a key research item in long-term follow-up, mainly included index segment degeneration, adjacent segment degeneration (ASD), heterotopic ossification (HO), or facet joint osteoarthritis.

In lumbar TDR surgery, the operation is conducted mainly by the anterior retroperitoneal approach, which may result in anterior longitudinal ligament dissection with an increased risk of vascular injury and post-sympathectomy syndrome. In addition, anterior revision surgery has a much greater risk of scarring around the vasculature from the primary procedure [33]. A lateral approach for lumbar TDR surgery may decrease the intrinsic weaknesses and complications of the anterior approach. As Tohmeh and Smith [42] described in their study, 89% of XL TDR surgery was performed using a left-side approach, and no intraoperative complications occurred during the 3-year postoperative follow-up. With a lateral approach, anterior longitudinal ligament can be preserved, the risk of vascular injury can be minimized, and performing TDR revision surgery is safer. However, the surgical approach does not completely explain the intraoperative complications. According to Mayer and Siepe [24] analysis, it is partly due to the learning curves of surgeons. As presented by Siepe et al. [39], although extensive knowledge has been gained since the first TDRs in the 1980s, the current data should still be considered as the “worst-case scenario,” including both the clinical and technical learning curves.

In the included studies, device-related complications mainly related to prosthesis subsidence and dislocation were reported in nine studies. Since the first commercial lumbar TDR prosthesis of the SB Charité was designed and used in a clinical setting, improvements in materials, design, and clinical experience have been constantly updated. For instance, the use of AcroFlex prostheses was terminated due to the high device failure rate during clinical application. David [7] reported six cases of device-related complications, including three cases of subsidence, two cases of early core subluxation, and one case of late core failure. Late core failure occurred 9.5 years after lumbar TDR surgery and might have been caused by core oxidation during sterilization of gamma. Therefore, with improvement in the sterilization technique, the Charité artificial disc dramatically reduced the chances of oxidation of the core. In the included studies, complications due to the TDR prosthesis itself are infrequent, and the failure of devices might be related to the surgeon’s unfamiliarity with the TDR surgical technique and the lack of strict patient selection criteria. In the Putzier et al. [34] study, 84 Charité artificial discs type I-III were implanted into 71 patients with a follow-up of 17 years. Although type I and type II had confirmed design drawbacks and were modified to type III, the overall clinical outcomes and radiological results in relation to these three types of prostheses were not significantly different. This finding may indicate that the prosthesis might not be the main cause of the complications.

ASD has been a common complication concern after lumbar TDR, especially in long-term follow-up. Siepe et al. [39] reported a 2.2% (4/181) incidence of ASD after a mean follow-up of 7.4 years, including adjacent disc herniation and symptomatic adjacent degeneration. However, it is uncertain whether ASD was caused by TDR surgery. Putzier et al. [34] reported a much higher incidence ASD of 17%, in which incident ASD was found only in cases with surgery segment spontaneous ankylosis or fusion after implant failure. In their study, the heterotopic ossification occurrence rate was 60%, and there were significant limitations in this study, such as undersized prostheses, suboptimal device placement, and bad candidate selection [21]. ASD prevention by lumbar TDR seems to be a more theoretical and biomechanical concept, and more clinical studies are needed to clarify the reasons for ASD.

HO is characterized by ossification around the prosthesis that impacts motion. The original design concept of TDR was to restore the intervertebral segment ROM and prevent adjacent levels degenerating. Without index level preservation, the lumbar TDR prosthesis will act as an interbody spacer, losing the function of preventing adjacent degeneration [44]. HO was reported in four studies, all related to the Charité prosthesis. David [7] reported a 6.6% incidence of ossification, four cases of partial ossification, and three cases of complete ossification, in which six of the seven occurred within 2 years of surgery. Tohmeh and Smith [42] reported three patients (3/29, 10.3% at 3 years) in which HO affected segmental ROM but with no ankylosis. Huang et al. [18] reported that patients with ROM of less than 5° in the lumbar region and TDR segments had a higher prevalence of adjacent level degeneration (ALD), suggesting that significant ROM after lumbar TDR may reduce the incidence of ALD. Cakir et al. [5] analyzed the interaction of clinical outcome with total lumbar ROM and showed that better clinical outcome was typical of patients with a higher total lumbar ROM preservation postoperatively. Putzier et al. [34] reported a much higher complication ossification rate of 60% in their retrospective clinical radiological study, where 32 patients had spontaneous ankylosis and impaired segmental mobility and were graded as type IV according to the McAfee classification [25]. However, there was no consensus regarding the reasons for the formation of the HO. For instance, David [7] considered that it might be related to delayed mobilization and active physiotherapy, while Putzier et al. [34] assumed that ossification resulted from degenerative changes to the segment-related tissue. Furthermore, the influence of HO on prognosis is often underestimated, as TDR requires ossification at the interface between the prosthesis and endplate, but this ossification can be progressive during follow-up. McAfee et al. [25] assumed that HO does not necessarily impact prosthetic movement; only severe ossification interferes with segmental motion. Therefore, HO did not necessarily indicate further restriction of TDR prosthetic movement.

Facet joint osteoarthritis observed in chronic low back pain patients is not a specific complication of TDR. The incidence of facet joint osteoarthritis is not uncommon, but its relationship with TDR is still controversial. The Van de Kelft et al. study of the 16% of patients with facet joint osteoarthritis suggested that inadequate restoration of physiological kinematics might induce facet joint degeneration. Tohmeh and Smith [42] proposed an anterior approach that might result in excised anterior longitudinal ligament and destabilization of segments and that, in turn, would increase the load on the facet joints. Another cause may be related to the design concepts of the total artificial disc prosthesis. Maverick and ProDisc are designed as semiconstrained prostheses, and the Charité disc is an unconstrained prosthesis. Regarding load translation, unconstrained prostheses share more load with the surrounding structures during the motion of the segments, which may increase facet contact forces [20]. Kim et al. reported a finite element model analysis of the Charité, ProDisc, and Maverick prostheses, finding that extension load could increase the operating facet pressure to a higher level than in adjacent segments; therefore, the risk of facet degeneration may be increased [22].

In a review of previous studies of lumbar TDR at 2-year follow-up, the reoperation rate ranged from 3.7 to 8.8% [3, 26, 40, 49]. Although the incidence of TDR was not significantly different compared with that of the fusion group at short-term follow-up, due to the surgical approach and complex local conditions, revision surgery was quite challenging, and the prognosis was not clear in the long run. As reported in this study, revision ranged from 0 to 39.3%, and as shown in Table 5, 8/13 studies had a revision rate of less than 10%. Only two studies by Meir et al. [27] and Laugesen et al. [20] had a revision rate of more than 30%, with possible causes related to TDR prosthesis design and patient selection criteria. According to the Siepe et al. [39] study, the indications for reoperations were classified into four groups: device- or technique-related reoperation (e.g., implant subsidence, implant dislocation, implant fracture, facet arthrosis), general surgery (e.g., incisional hernia, retroperitoneal hematoma), persisting symptoms of low back pain, or adjacent segment pathologies (e.g., adjacent degenerative spondylolisthesis, disc hernia).

Reoperation surgery is quite complex, and the surgical technique to be used should be decided on an individual basis. A previous study showed that some asymptomatic changes, such as migration and subsidence, can be treated with conservative treatment alone [32]. Symptomatic patients with persistent pain, neurologic symptoms, or other symptoms had poorer quality of life after lumbar surgery and might need reoperation. Strategies for reoperation [2] mainly involve three methods: first, the insertion of a posterior dynamic implant or elimination using supplemented fixation; second, the use of a new prosthesis; and third, removal of the prosthesis with anterior interbody fusion. However, another affected reoperation outcome is the reoperation approach. Anterior revision procedures for lumbar TDR are more likely to increase the risk of complications. The far-lateral or transpsoas approach is recommended to reduce this risk. In the study of Line A by Laugesen et al. [20], a comparison of the clinical outcome of patients with or without revision surgery found no significant difference between the two groups, except for back pain score.

A limitation of this study was that the search strategy in this article used only the PubMed database; a comprehensive literature search might need to be performed to obtain a more reliable conclusion. In this study, only three RCTs met the inclusion criteria, and it was not sufficient to conclude that TDR was preferred for fusion surgery in treating degenerative disc diseases. Therefore, more high-quality RCTs are needed to confirm the efficiency and safety of lumbar TDR.

## Conclusions

This study showed that lumbar TDR effectively resulted in pain relief and an improvement in quality of life at mid- to long-term follow-up. Complication and reoperation rates were acceptable, although improved surgical technique and an optimized prosthesis design are required to further improve clinical outcomes. However, this study did not provide sufficient evidence to support the argument that lumbar TDR is superior to fusion surgery. To answer that question, a greater number of high-quality RCTs are needed.

## Abbreviations

ALD: Adjacent level degeneration
AMSTAR: Assessment of Multiple Systematic Reviews
ASD: Adjacent segment degeneration
HO: Heterotopic ossification
NIS: Nationwide inpatient sample
ODI: Oswestry disability index
RCTs: Randomized controlled trials
TDR: Total disc replacement
VAS: Visual analogue scale
